# Adenosine as a modulator of human islet function and hypoxic tolerance

**DOI:** 10.3389/fbioe.2026.1828405

**Published:** 2026-06-01

**Authors:** Quentin Perrier, Adam Jones, Timothy Sganga, Lori Byers, Thomas Ritchey, Sandrine Lablanche, Christopher Fraker, Kelsey H. Fisher-Wellman, Alice A. Tomei, Rita Bottino, Giuseppe Orlando, Amish Asthana

**Affiliations:** 1 Univ. Grenoble Alpes, INSERM U1055, Department of Pharmacy, Grenoble Alpes University Hospital, LBFA, Grenoble, France; 2 Virginia Tech-Wake Forest School of Biomedical Engineering and Sciences, Winston Salem, NC, United States; 3 Wake Forest Institute for Regenerative Medicine, Wake Forest University School of Medicine, Winston Salem, NC, United States; 4 Department of Surgery, Section of Transplantation, Atrium Wake Forest Baptist Medical Center, Winston Salem, NC, United States; 5 Univ. Grenoble Alpes, INSERM U1055, Department of Diabetology, Endocrinology and Nutrition, Grenoble Alpes University Hospital, LBFA, Grenoble, France; 6 Diabetes Research Institute, University of Miami Miller School of Medicine, Miami, FL, United States; 7 Department of Cancer Biology, Wake Forest University School of Medicine, Winston Salem, NC, United States; 8 Imagine Islet Center, Imagine Pharma, Pittsburgh, PA, United States

**Keywords:** cytoprotection, islet transplantation, metabolic suppression, oxidative stress, reperfusion injury, type 1 diabetes, β-cell replacement

## Abstract

Islet transplantation hold promises as a cell therapy for managing type 1 diabetes, but its effectiveness remains limited due to early graft loss caused by hypoxia and associated stressors. Identifying strategies to enhance islet resilience is imperative for improving clinical outcomes. We hypothesized that mitigating this injury requires shifting focus from augmenting oxygen supply to reducing the cellular metabolic demand. Adenosine (AD), an endogenous purine nucleoside with immunomodulatory and metabolic regulatory roles, emerges as a candidate to mitigate these challenges. This study evaluates the impact of AD on human islet (HI) viability, function, and metabolic suppression under hypoxic stress (1% O_2_). Results demonstrated that AD at 1 mM induced a transient reduction in insulin content without impairing viability or functional indices (stimulation index, insulin secretion). These changes were reversible within 96 h of withdrawal. Preconditioning HI with 1 mM AD prior to 48-h hypoxia preserved both viability and glucose-stimulated insulin secretion, mitigating functional losses up to 48 h post-reperfusion. These findings establish AD preconditioning as an effective, reversible approach to reduce islet metabolic demand and enhance hypoxia tolerance during the early post-transplantation period. Furthermore, AD can be readily integrated into various stages of beta-cell replacement workflow, including islet culture, encapsulation, and 3D bioprinting. Overall, AD-mediated metabolic downregulation offers a novel strategy for mitigating ischemia-reperfusion injury, paving the way for more efficient and resilient cell-based therapies for beta-cell replacement.

## Introduction

The clinical success of cellular therapies is inherently constrained by the extensive metabolic insults that transplanted constructs endure during the initial engraftment period. In the context of type 1 diabetes (T1D), the allotransplantation of pancreatic islets has emerged as a minimally invasive alternative to whole-pancreas transplantation (Wojtusciszyn et al., 2019; Perrier et al., 2021), capable of improving glycemic control and reducing mortality in patients with particularly unstable disease (Lablanche et al., 2018; Perrier et al., 2025). However, this therapeutic potential is substantially undermined by extensive early graft attrition, wherein a significant fraction of the islet biomass is irretrievably lost within the initial hours to days post-transplantation (Holendová et al., 2024). This acute loss is driven by a convergence of inflammatory, oxidative, and biochemical stressors that are further exacerbated by a critical underlying driver of cell death: severe hypoxia (Toso et al., 2005). Islets account for only 1%–2% of pancreatic volume yet receive 5%–10% of its blood flow (Jansson et al., 2016). This intrinsically high metabolic demand and the disruption of intra-islet capillary network during the harsh isolation process render them especially vulnerable to oxygen deprivation. Following transplantation, the lag in revascularization can extend this vulnerability, leading to necrosis and apoptosis within the graft (Ke and Murphy, 2019). Consequently, extensive research efforts have been focused on mitigating this hypoxic burden. Prevailing strategies have centered on augmenting oxygen delivery via synthetic carriers (i.e., perfluorocarbons), (Lee et al., 2018), or via oxygen-generating biomaterials (Pedraza et al., 2012). Other approaches have sought to accelerate neovascularization through the localized delivery of pro-angiogenic factors, including vascular endothelial growth factor (VEGF) and platelet-derived growth factor (PDGF) or by co-transplanting islets with endothelial cells, endothelial progenitor cells, or microvascular fragments (Grunewald et al., 2006; Kalka et al., 2000; Yang et al., 2002). Despite their conceptual merit, these supply-side strategies have proven insufficient in preserving the viability of clinically relevant graft volumes, thus highlighting a compelling need for alternative therapeutic paradigms (Ness and Cushing, 2007; Harrison et al., 2007).

A potentially synergistic yet underexplored strategy involves shifting the therapeutic focus from augmenting oxygen supply to reducing metabolic demand. Pharmacological induction of a reversible state of metabolic quiescence may represent a complementary “demand-side” strategy. By transiently suppressing islet metabolism before and immediately after implantation, their oxygen requirements can be lowered, potentially extending their survival during the avascular phase. This concept of inducing a state of reversible metabolic dormancy remains a nascent and largely uninvestigated therapeutic avenue. Adenosine (AD), an endogenous purine nucleoside, serves as a key regulator of cellular metabolism and exhibits potential immunomodulatory properties (Antonioli et al., 2015). Foundational studies with rodent islets established that AD can suppress glucose-stimulated insulin secretion and islet metabolism, predominantly through A1 receptor activation (Ismail et al., 1977; Szkudelski and Szkudelska, 2015). However, the profound architectural, cellular, and physiological differences between rodent and human islets (HI), including distinct cellular compositions and paracrine regulatory networks preclude the direct extrapolation of these findings (Bosco et al., 2010; Cottet-Dumoulin et al., 2023). Furthermore, the response of HI to AD, particularly within the context of hypoxic stress, has yet to be systematically elucidated.

Taken together, we hypothesized that AD could be leveraged to induce a state of controlled, reversible metabolic suppression in HI, thereby conserving the islet’s cellular energy and enhancing their tolerance to the hypoxic conditions’ characteristic of the early post-transplantation period. Herein, we have characterized the dose-dependent effects of AD on HI viability, secretory function, and glucose responsiveness. We have further delineated the kinetics of functional recovery following AD withdrawal and, tested the central hypothesis that AD pre-conditioning can confer robust cytoprotection against hypoxia-induced injury. Validation of this approach would position AD as a novel, readily translatable adjunct to beta-cell replacement therapy, spanning applications from *ex vivo* culture of cadaveric islets to biofabrication strategies such as 3D printing, as well as conditioning of stem cell-derived islet-like clusters prior to their implantation in micro- and macroencapsulation devices (Miller et al., 2025).

## Methods

### Biological materials

HI were obtained from Imagine Pharma® (Pittsburgh, Pennsylvania, United States) 1 day after their isolation from cadaveric donor pancreatic tissue. Upon receipt, HI were cultured for 24 h in PIM(R)® medium (Recovery Medium, Prodo Laboratories Inc., Aliso Viejo, CA, United States) at 37 °C in a humidified atmosphere containing 5% CO_2._ They were then transferred to PIM(S)® medium (standard media, no AD inside, Prodo Laboratories Inc.) for an additional 24 h prior to experimentation.

### AD treatment

HI were treated with AD (Sigma-Aldrich), which was added directly to the PIM(S) culture medium at indicated concentrations. After 24 h of exposure, HI were washed with PBS and transferred to fresh PIM(S) medium lacking AD for subsequent analysis.

### Hypoxia exposure

Hypoxia was induced using the ExVivo GMP System X2® (Biospherix, Parish, NY, United States). Islets were incubated in pre-conditioned hypoxic medium at 37 °C, in an atmosphere containing 1% O_2_, and 5% CO_2_ for 48 h. Afterward, reperfusion was initiated by replacing the hypoxic medium with fresh normoxic PIM(S) under normoxic conditions (21% O_2_), and analyses were performed at 2, 24, or 48 h post-oxygenation. This protocol was designed to mimic *in vivo* ischemia-reperfusion injury.

### Viability assay

Islet viability was assessed using confocal microscopy on whole, intact HI stained with 1 µM Syto13 and 10 μg/mL propidium iodide (PI) (Thermo Fisher Scientific). Excitation was achieved using the 488 nm and 561 nm lasers. The emission spectra were collected between 500 and 600 nm for Syto13 and 590–690 nm for PI. Images were acquired using an Olympus IX83 inverted confocal microscope (OLYMPUS FluoView FV300, Center Valley, PA, United States). Z-stacks were collected using a 10 μm step size and a pinhole of 1 (Airy units) for all channels. Quantification was done with a macro on ImageJ software (version 8) as previously described (Perrier et al., 2024). Results were expressed as Viability % = 100 * number of green fluorescent cells/(total number of green and red cells), with 50 IEQ analyzed per condition.

We evaluated two sets of time points: recovery time points following AD withdrawal (no hypoxia): D0 (0 h), D2 (48 h), and D4 (96 h); and reperfusion time points following hypoxia exposure: R2 (2 h), R24 (24 h), and R48 (48 h).

### Glucose stimulated insulin secretion (GSIS) assay

GSIS was performed under static conditions using Krebs-Ringer bicarbonate buffer (KRB, 115 mM NaCl, 4.72 mM KCl, 2.56 mM CaCl2, 1.2 mM KH2PO4, 1.2 mM MgSO4, 3.89 mM NaHCO3, 24.4 mM HEPES, pH 7.4) supplemented with 0.2% bovine serum albumin (BSA) (w/v). Islets were first rinsed in low-glucose KRB (2.8 mM) and then pre-incubated for 1 h at 37 °C in the same low glucose solution. Samples were then subjected to sequential incubations – LG1: 1 h in 2.8 mM glucose (baseline); HG – 1 h at in high-glucose KRB (16.7 mM); and LG2: 1 h in 2.8 mM glucose (return to baseline). Following incubation, insulin content was assessed after overnight incubation at −20 °C in ethanol acid solution (375 mL absolute ethanol + 7.5 mL 12.7 M HCl + 117.5 mL distilled water). Supernatants corresponding to LG1, HG, LG2 and insulin content were analyzed for insulin using a Mercodia ELISA (Winston-Salem, North Carolina, United States) and quantified using a SpectraMax plate reader (Molecular Devices, San Jose, CA, United States). Data were normalized to the total insulin content. The stimulation index (SI) was determined by the ratio of insulin secretion in HG to that in first LG (HG/LG1), while the recovery index (RI) was calculated as the ratio of insulin secretion in HG to that in second LG (HG/LG2). The GSIS delta was calculated with the normalized insulin secretion value: Δ = (HG − LG1). Each GSIS condition used 100 IEQ, and functional assays were conducted at the same time points as viability assessments.

### Alamar blue assay

Metabolic activity was assessed using the Alamar Blue assay (resazurin-based, ThermoFisher Scientific). Briefly, islets (200 IEQ) were incubated with Alamar Blue reagent diluted in PIMS medium (10% v/v) for 4 h at 37 °C in a humidified atmosphere with 5% CO_2_. The reduction of resazurin to resorufin by metabolically active cells was quantified by measuring fluorescence (excitation: 544 nm; emission: 590 nm) using a SpectraMax plate reader (Molecular Devices). Results were normalized to control wells and expressed as a percentage of control viability. This assay provides a quantitative assessment of the overall cellular reductive metabolic capacity, reflecting the energy demands of the islet.

### Statistical analyses

Data were expressed as mean ± standard error of the mean (SEM), and the normality of variables has been investigated using the Shapiro–Wilk test. The number of independent experiments and the number of total replicates is indicated as N and n, respectively, in figure legends. All statistical tests were performed by GraphPad (version 8.0.1). Comparisons among groups were made using one-way ANOVA (or an appropriate non-parametric equivalent), with significance defined as p < 0.05.

## Results

### Optimization of AD concentration for HI treatment

**FIGURE 1:**
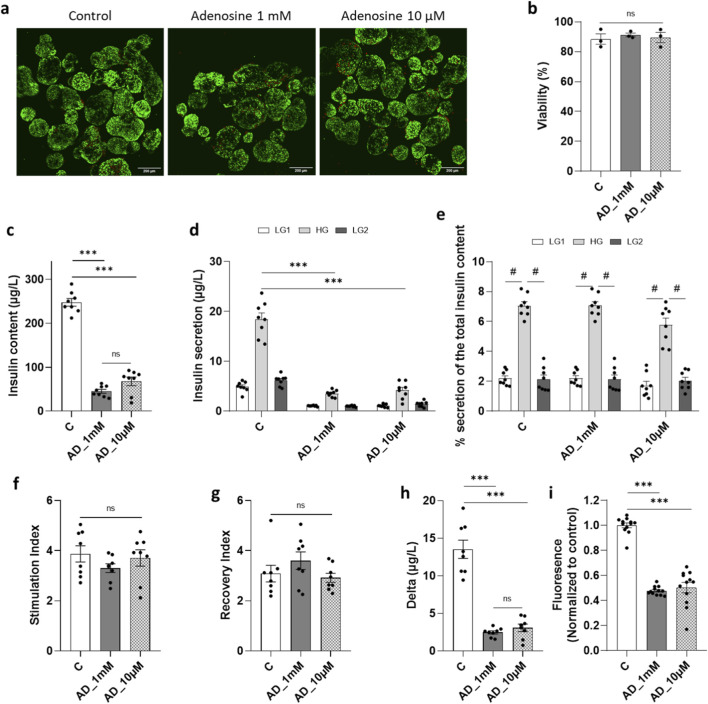
Dose effect of AD on HI viability and functionality. HIs were treated with 1 mM or 10 µM AD for 24 h. **(a)** Representative confocal images showing viability staining using Syto13 (green; live cells) and PI (red; dead cells) (N = 3 HI donors; no technical replicates). **(b)** Quantification of HI viability (% live cells). To conduct GSIS, 100 IEQ were exposed to subsequent glucose concentrations in low glucose (LG1, 2.8 mM), high glucose (HG, 16.7 mM) and LG2 (2.8 mM) solutions. (N = 3 HI donors; n = 8–12 total replicates). **(c)** Insulin content obtained at the end of the GSIS with the exposure to ethanol acid solution. **(d)** Insulin secretion (µg/L). **(e)** Insulin secretion normalized to total insulin content. **(f)** GSIS stimulation index. **(g)** GSIS recovery index. **(h)** GSIS delta of insulin secretion (HG-LG). **(i)** Overall metabolic activity estimated with fluorescence normalized to the control condition from an Alamar blue assay. Data are represented as mean ± SEM, One-way ANOVA with Tukey *post hoc* test, ***p < 0.001, #p < 0.001, ns, not significant; AD, adenosine; C, control.

The viability of HI treated for 24 h with 1 mM (91.2% ± 1.3%) or 10 µM AD (89.5% ± 3.4%) was comparable to that of the control group (88.5% ± 3.4%; p = 0.809; Figures 1a,b), indicating that AD was not cytotoxic at either concentration. Moreover, confocal images showed no apparent changes in islet integrity or morphology. The 1 mM and 10 µM AD groups exhibited insulin content levels of 45.4 ± 4.4 μg/L and 68.0 ± 9.7 μg/L, respectively, both significantly lower than the control (247.7 ± 8.6 μg/L; p < 0.001; Figure 1c). Similarly, insulin secretion in response to high glucose was significantly reduced in both AD-treated groups (1 mM: 3.5 ± 0.2 μg/L; 10 µM: 4.2 ± 0.6 μg/L) compared to control (18.5 ± 1.2 μg/L; p < 0.001; Figure 1d). This trend was also reflected in the delta values (HG-LG1, Figure 1h). Across all conditions, the relative amount of insulin secreted (normalized by total insulin content) in response to HG, was significantly higher than that under the two-glucose conditions (LG1 and LG2, p < 0.001, Figure 1e), suggesting preserved glucose responsiveness. Furthermore, no significant differences were observed in the SI (control: 3.87 ± 0.32; AD 1 mM: 3.30 ± 0.18; AD 10 µM: 3.68 ± 0.32; p = 0.364; Figure 1f) and the RI (control: 3.09 ± 0.33; AD 1 mM: 3.60 ± 0.35; AD 10 µM: 2.92 ± 0.18; p = 0.261; Figure 1g). The overall metabolic activity estimated by the Alamar blue assay results was decreased in both conditions (1 mM and 10 µM) compared to the control (Figure 1i, p < 0.001). This reduction in metabolic activity is a necessary precursor for preserving cellular energy during subsequent periods of oxygen deprivation. The 1 mM AD condition was selected for subsequent experiments due to its significantly and consistently lower data variability across all functional and metabolic assays. Specifically, the coefficient of variation (CV%) for the 1 mM AD group was found to be lower than the 10 µM AD group for all parameters measured: insulin secretion results (15% vs. 25%), insulin content (27% vs. 40%), insulin secretion in high glucose (19% vs. 41%), and Alamar blue metabolic activity (7.2% vs. 28.5%)

### Recovery of HI post-AD treatment

**FIGURE 2:**
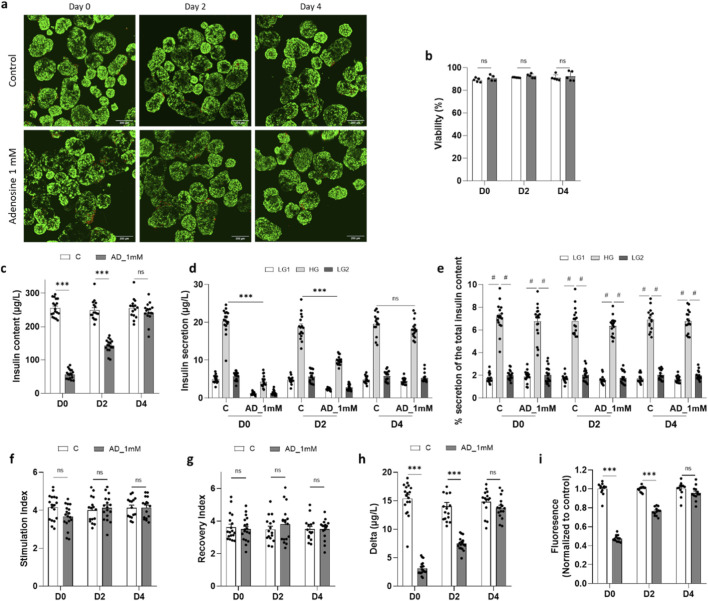
Recovery of HI following withdrawal of AD. HIs were treated with 1 mM AD for 24 h and then analyzed immediately (D0), 48 h (D2), and 96 h (D4) post-treatment withdrawal. **(a)** Representative confocal viability images using Syto13 (green; live cells) and PI (red; dead cells) (N = 5 HI donors; no technical replicates). **(b)** Quantification of HI viability (% live cells). To conduct GSIS, 100 IEQ were exposed to subsequent glucose concentrations in low glucose (LG1, 2.8 mM), high glucose (HG, 16.7 mM) and LG2 (2.8 mM) solutions. (N = 5 HI donors; n = 15–18 total replicates). **(c)** Insulin content obtained at the end of the GSIS with the exposure to ethanol acid solution. **(d)** Insulin secretion (µg/L). **(e)** Insulin secretion normalized to total insulin content. **(f)** GSIS stimulation index. **(g)** GSIS recovery index. **(h)** GSIS delta of insulin secretion (HG-LG). **(i)** Overall metabolic activity estimated with fluorescence normalized to the control condition from an Alamar blue assay (N = 3 HI donors; n = 12 replicates). Data are represented as mean ± SEM, One-way ANOVA with Tukey *post hoc* test, ***p < 0.001, #p < 0.001, ns, not significant; AD, adenosine; C, control; D, day.

To assess the reversibility of the metabolic effects of AD (1 mM) treatment, HI viability and functionality were evaluated at D0 (0 h), D2 (48 h), and D4 (96 h) following AD withdrawal. The viability of control and AD-treated HI was comparable at all time points (D0: 89.1% ± 0.8% vs. 90.8% ± 1.1%, p = 0.891; D2: 91.3% ± 0.1% vs. 92.7% ± 0.8%, p = 0.949; and D4: 91.1% ± 1.2% vs. 92.5% ± 1.9%, p = 0.946; Figures 2a,b). The insulin content of AD-treated HI was significantly lower than that of the control at D0 (57.1 ± 3.3 μg/L vs. 255.6 ± 6.1 μg/L, p < 0.001) and D2 (142.6 ± 4.7 μg/L vs. 249.0 ± 7.4 μg/L, p < 0.001), but was comparable at D4 (242.9 ± 7.4 μg/L vs. 254.9 ± 7.9 μg/L, p = 0.898, Figure 2c), indicating complete reversibility of AD’s suppressive effect on insulin biosynthesis (insulin content recovery post AD treatment of 22% at D0, 57% at D2, and 95% at D4). A similar trend was observed in the level of insulin secreted in response to HG, which was significantly lower in the AD-treated group at D0 (4.3 ± 0.3 μg/L vs. 20.4 ± 0.8 μg/L, p < 0.001) but comparable at D4 (18.2 ± 0.6 μg/L vs. 19.6 ± 0.7 μg/L, p = 0.317, Figure 2d). This trend was also reflected in the delta values (Figure 2h) and the overall metabolic activity (Figure 2i). Across all time points, the relative amount of insulin secreted (normalized by total insulin content) in response to HG, was significantly higher than that under the two low-glucose conditions, for both the conditions (p < 0.001, Figure 2e). No significant differences were observed in the SI (p = 0.339, Figure 2f) or the RI (p = 0.675, Figure 2g) between the control and AD-treated HI at any time point. These results supposed that AD-induced functional suppression is transient, reversible and does not impair the intrinsic glucose-sensing capacity and metabolic activity of HI.

### Protective effect of AD preconditioning on HI following hypoxic exposure

**FIGURE 3:**
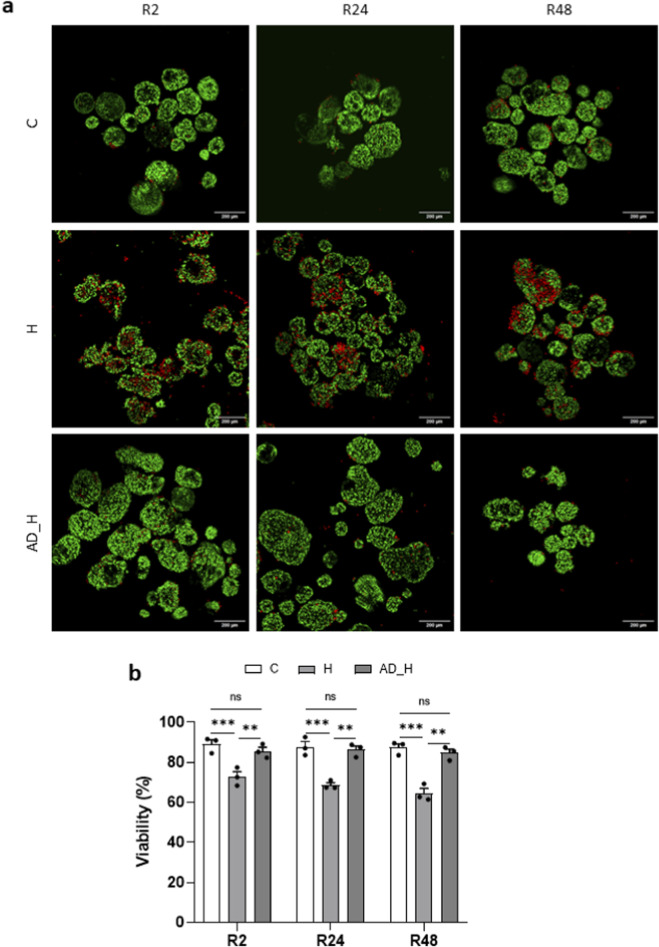
Protective effect of AD preconditioning on HI viability following hypoxia. HIs were preconditioned with 1 mM AD for 24 h and then subjected to 48 h of hypoxia (1% O_2_), followed by 2 h (R2), 24 h (R24), or 48 h (R48) of reperfusion in normoxic medium (21% of O_2_). **(a)** Representative confocal viability images using Syto13 (green; live cells) and PI (red; dead cells). **(b)** Effect of adenosine before hypoxia exposure on HI viability (N = 3 HI donors; no technical replicates). Data are represented as mean ± SEM, Scale bars = 200 μm, One-way ANOVA with Tukey *post hoc* test, **p < 0.01, ***p < 0.001, ns, not significant; AD_H, AD treatment for 24 h followed by 48 h of hypoxia; H, hypoxia for 48 h; C, control (normoxic without AD); R, reperfusion.

HI were exposed to 48 h of hypoxia (1% O2), followed by normoxic incubation (reperfusion) for 2, 24, or 48 h (R2, R24 and R48, respectively). Hypoxia led to a significant reduction in viability (−16.1% to −22.8% of viability) at all time points when compared to non-hypoxic control (R2: 72.9% ± 2.6% vs. 89.0% ± 2.3%, p < 0.001; R24: 68.7% ± 1.2% vs. 87.7% ± 2.6%, p < 0.001; and R48: 64.6% ± 2.4% vs. 87.4% ± 1.9%, p < 0.001; Figures 3a,b). HI preconditioned with 1 mM AD for 24 h prior to hypoxia maintained significantly higher viability than the non-preconditioned HI at all reperfusion time points (p < 0.001; Figure 3b), with values comparable to control (R2: 85.6% ± 2.0%, p = 0.504; R24: 86.3% ± 1.9%, p = 0.886; R48: 84.7% ± 1.9%, p = 0.656; Figure 3b).

**FIGURE 4:**
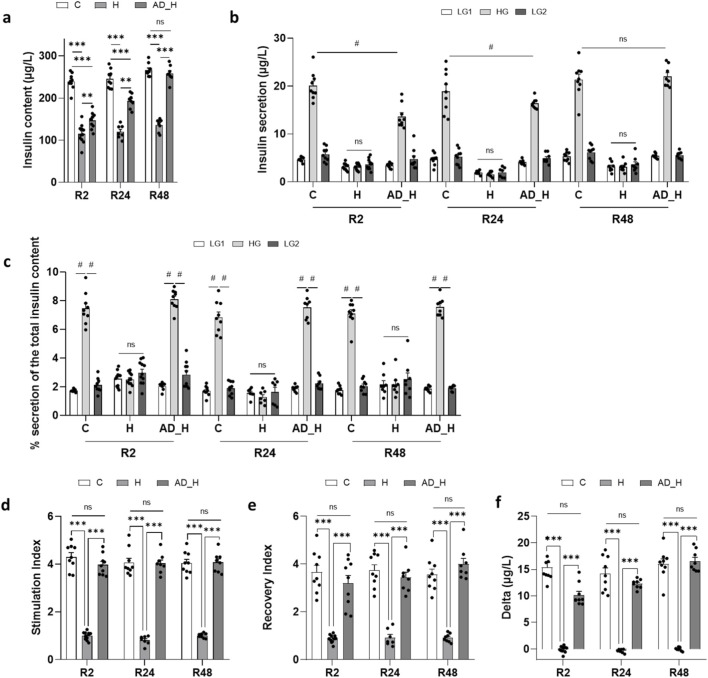
Protective effect of AD preconditioning on HI functionality following hypoxia. HIs were preconditioned with 1 mM AD for 24 h and then subjected to 48 h of hypoxia (1% O_2_), followed by 2 h (R2), 24 h (R24), or 48 h (R48) of reperfusion in normoxic medium (21% of O_2_). To conduct GSIS, 100 IEQ were exposed to subsequent glucose concentrations in low glucose (LG1, 2.8 mM), high glucose (HG, 16.7 mM) and LG2 (2.8 mM) solutions. (N = 3, number of different HI donors used and n = 7–11, number of total replicates). **(a)** Insulin content obtained at the end of the GSIS with the exposure to ethanol acid solution. **(b)** Insulin secretion (µg/L). **(c)** Insulin secretion normalized to total insulin content. **(d)** GSIS stimulation index. **(e)** GSIS recovery index. **(f)** GSIS delta of insulin secretion (HG-LG). Data are represented as mean ± SEM, One-way ANOVA with Tukey *post hoc* test, *p < 0.05, **p < 0.01, ***p < 0.001, #p < 0.001, ns, not significant. AD_H, AD treatment for 24 h followed by 48 h of hypoxia; H, hypoxia for 48 h; C, control, R, reperfusion.

Hypoxia also severely disrupted both insulin biosynthesis and secretion. The insulin content in hypoxia-only HI was significantly lower compared to both control and AD-preconditioned HI at all time points (p < 0.01; Figure 4a). The insulin content of AD-preconditioned HI was significantly lower than control at D0 (146.5 ± 6.8 μg/L vs. 239.3 ± 6.5 μg/L; p < 0.001), but showed partial restoration at 24 h post reperfusion (193.3 ± 6.0 μg/L vs. 245.2 ± 7.3 μg/L; p < 0.001), with full recovery at 48 h post reperfusion (258.2 ± 6.6 μg/L vs. 266.3 ± 5.0, p = 0.993; Figure 4a). A similar trend was observed in the level of insulin secreted in response to HG. Hypoxia-only HI lost glucose responsiveness, exhibiting no significant difference across LG1, HG, and LG2 (Figures 4b,c). In contrast, AD-preconditioned HI maintained robust insulin secretion at D0 (13.6 ± 0.8 μg/L vs. 20.1 ± 1.0 μg/L, p < 0.001) and were fully comparable to control by D4 (22.0 ± 0.7 μg/L vs. 21.3 ± 1.2, p = 1; Figure 4b). Furthermore, this pattern was also reflected in the delta values (Figure 4f) and normalized secretion profiles (Figure 4c), confirming that glucose responsiveness was preserved.

Finally, functional indices further emphasized the protective effect of AD. Hypoxia drastically reduced SI at all reperfusion time points (R2: 1.00 ± 0.05 vs. 4.30 ± 0.18, p < 0.001; R24: 0.81 ± 0.07 vs. 4.07 ± 0.19, p < 0.001; R48: 1.01 ± 0.04 vs. 4.05 ± 0.16, p < 0.001; Figure 4d). AD-preconditioned HI retained functional SI, which remained significantly higher than that of hypoxia-only HI (p < 0.001) and comparable to control at all time points (R2: 3.97 ± 0.15, p = 0.193; R24: 4.03 ± 0.15, p = 0.985; R48: 4.07 ± 0.15, p = 0.991, Figure 4d), confirming intact glucose sensing. A similar trend was observed for the RI, with hypoxia-only HI exhibiting a significant decrease (p < 0.001), but AD-preconditioned HI being comparable to control (p > 0.05; Figure 4e), further suggesting that AD mitigates both viability loss and functional decline under hypoxic conditions. Furthermore, the same trend of higher viability and functional preservation was observed after 5 days of reperfusion (Supplementary Figure S1), demonstrating that the early AD-mediated protection prevented the irreversible functional decline experienced by non-preconditioned HI, which failed to recover even by this later time point.

## Discussion

The widespread clinical application of pancreatic islet transplantation is impeded by extensive early graft loss, largely driven by severe hypoxia during the avascular post-transplantation period (Toso et al., 2005). This study tested the hypothesis that inducing reversible metabolic suppression using AD could enhance HI tolerance to hypoxic stress. We have demonstrated for the first time that a 24 h pre-conditioning with 1 mM AD is safe, fully reversible, and confers significant protection against hypoxia-induced islet death and dysfunction. These results validate a “demand-side” metabolic modulation strategy as a potent approach to augment the resilience of cellular therapies.

AD is not a new compound in transplantation; in fact, it has long been used in organ preservation. Notably, AD is a key component of the University of Wisconsin (UW) preservation solution, widely used in pancreas and solid organ transplantation. In this context, AD contributes to ATP replenishment and maintenance of cellular energy homeostasis during cold ischemia. However, UW solution is applied to the preservation of the whole pancreas, meaning that AD exposure occurs at the organ level rather than being specifically targeted to islets. Moreover, this exposure is limited to the relatively short duration of cold storage before islet isolation. In contrast, our approach uses AD during *ex vivo* culture of isolated human islets, enabling more controlled, direct, and reversible modulation of islet metabolic demand. These distinctions suggest that AD-based strategies could be complementary across different stages of the islet transplantation workflow.

A fundamental prerequisite for any preconditioning agent is efficacy, safety, and complete reversibility. Our initial experiments demonstrated that AD induced a transient suppression of insulin biosynthesis and secretion, without compromising viability or glucose responsiveness (Figures 1a–e). Despite the reduced metabolic activity (Figure 1i) and insulin output, the stimulation and recovery indices remained unaffected, suggesting that AD modulates metabolic activity without impairing glucose-sensing mechanisms (Figures 1f,g). Overall, this decoupling of insulin content from secretion supports the notion of controlled metabolic downregulation rather than cellular dysfunction. While the regulatory effects of AD on metabolism have been studied in rodent models, with micromolar concentrations inhibiting insulin secretion, (Ismail et al., 1977), its impact on HI has remained unexplored. Given the significant architectural and physiological differences between rodent and HI, the demonstration that both micromolar (10 µM) and millimolar (1 mM) concentrations of AD elicit a potent and safe metabolic downregulation in HI is a crucial validation. While the 1 mM concentration was selected for subsequent experiments due to its significantly lower data variability, statistical robustness, and supportive findings in hypoxia-exposed muscle cells, (Ke et al., 2021), the observed efficacy of the 10 µM concentration (Figure 1) suggests that a lower, potentially more clinically favorable dose is effective. Future translational work may focus on optimizing the 10 µM dose to minimize potential off-target effects. Furthermore, AD’s effects on both insulin content and secretion, and the overall islets metabolic activity were completely reversed within 96 h of withdrawal, reinforcing its suitability as a transient preconditioning agent (Figures 2a–e). This reversibility is critical for translational applications, as it ensures that while the islets are protected by reduced demand during the initial high-risk hypoxic window, they return to full functionality (with insulin content reaching 57% recovery by 48 h and full recovery by 96 h) once engraftment is underway.

The central finding of this investigation is the significant cytoprotective effect conferred by AD preconditioning against a severe hypoxic insult. HI subjected to 48 h of hypoxia (1% O_2_) without AD preconditioning exhibited a substantial loss of viability and a complete loss of glucose responsiveness that did not recover post-reperfusion (Figures 3b, 4). In contrast, AD-pre-conditioned HI maintained viability and preserved functional output, with stimulation indices comparable to controls even after 48 h of reperfusion (Figures 3b, 4d,e). It can be postulated that AD mitigates hypoxic injury by reducing islet metabolic demand, which is supported by reduced insulin biosynthesis (Figure 4a) and overall metabolic activity (Figure 1i). This reduction mitigates the cellular energy deficit, averting the subsequent cytotoxic cascades initiated by a severe oxygen-supply mismatch (Holendová et al., 2024). This protective phenomenon has been documented in other tissues, including skeletal muscle (Ke et al., 2021; Kim et al., 2014), heart (Sommerschild and Kirkebøen, 2000) and kidney transplantation (Lee et al., 2004), lending further credence to its potential as a broad-spectrum cytoprotective agent.

The regulatory effects of AD have been previously recognized in various biological processes, including their influence on somatotropic hormones (prolactin, growth hormone) (Dorflinger and Schonbrunn, 1985), inflammation, and glucose/lipid metabolism (Koupenova and Ravid, 2013). Mechanistically, the observed outcomes are congruent with the known action of AD via the A1 receptor (A1R). The binding of AD to A1R is proposed to activate inhibitory G proteins, which result suppression of adenylate cyclase activity, leading to a decrease in intracellular cAMP, and reduction in protein kinase A (PKA) activity, stabilization of K_ATP_ channel opening, membrane hyperpolarization and lower intracellular Ca^2+^ (Koupenova and Ravid, 2013; Zywert et al., 2011; Nichols et al., 1996). This signaling cascade ultimately culminates in reduced insulin exocytosis in rodents islets (Hillaire-Buys et al., 1987; Johansson et al., 2007). However, due to fundamental differences between rodent and HI, future investigations into receptor-specific pathways are warranted to unequivocally confirm this mechanism in HI and to further delineate downstream effects on oxygen consumption rates and ATP homeostasis.

This study represents a significant conceptual advance for the field, positioning metabolic preconditioning as a potential alternative to prevailing oxygen-supplementing “supply-side” strategies (e.g., perfluorocarbons, angiogenic factor release). While valuable, such strategies present considerable technical hurdles and potential risks, including reactive oxygen species overproduction (Lee et al., 2018). The “demand-side” approach established here offers a comparatively simple, effective, and highly translatable strategy that could also be employed synergistically with existing technologies. AD preconditioning could be seamlessly integrated into clinical islet transplantation protocols by supplementing the islet culture medium following isolation and prior to transplantation. Furthermore, this approach is exceptionally well-suited for tissue engineered islet grafts, including incorporation of AD into bioinks for 3D bioprinting of pancreatic constructs or loading into encapsulation devices to provide localized, sustained metabolic suppression during the critical initial phase of engraftment. Furthermore, this strategy might also prove advantageous in poorly vascularized implantation sites, where hypoxic burden is particularly high. Moreover, this metabolic modulation framework could be translatable to stem cell-derived islet-like clusters, which face similar challenges during engraftment and represent the next frontier in cellular therapy for T1D.

Importantly, the robust *in vitro* cytoprotection achieved through AD-mediated metabolic suppression in primary HI provides a critical foundation for the clinical translation of this novel demand-side strategy. However, several key challenges must be addressed to fully validate its therapeutic relevance. First, while the 48 h hypoxia model at 1% O_2_ represents a highly stringent and widely accepted surrogate of acute ischemic injury, it primarily captures the early hypoxic phase following transplantation. In clinical settings, transplanted islets may experience a prolonged period of compromised oxygenation lasting up to 10–15 days prior to full engraftment and revascularization. Future *in vitro* studies will therefore need to optimize the AD preconditioning regimen. Importantly, the relatively high concentration of AD (1 mM) used in this study suggests that, beyond classical receptor-mediated signaling, intracellular mechanisms may also contribute to the observed effects. AD can be transported into cells via equilibrative nucleoside transporters, where it may influence intracellular AMP/ATP balance and metabolic pathways. Such intracellular actions could contribute to the observed reduction in insulin biosynthesis and overall metabolic activity. A potential limitation of this approach relates to the pleiotropic nature of AD signaling. Off-target effects may include modulation of immune responses, vascular tone, and paracrine interactions within the islet microenvironment. Furthermore, at higher concentrations, non-specific intracellular metabolic effects cannot be excluded. Several strategies could mitigate these risks, including the use of lower, receptor-selective concentrations (e.g., micromolar range), transient exposure protocols as implemented here, and localized delivery approaches (e.g., encapsulation systems or biomaterials) to restrict systemic exposure. Moreover, other parameters should be optimized, including the evaluation of longer preconditioning durations and the potential benefit of maintaining continuous AD exposure, in order to sustain cytoprotection throughout this extended hypoxic window. Second, *in vivo* validation in relevant rodent models of diabetes is essential to confirm the translational potential of this approach. In particular, transplantation into streptozotocin (STZ)-induced diabetic immunodeficient mice will allow assessment of glycemic control and functional graft efficacy, while transplantation into non-diabetic recipients may help isolate early engraftment (fast vascularization, early C-peptide detection) and survival dynamics independent of metabolic demand. Such approaches will be critical to determine whether AD-mediated metabolic suppression translates into improved graft function and restoration of normoglycemia *in vivo*.

Together, these next steps will be pivotal in establishing AD preconditioning as a clinically actionable strategy for improving islet graft survival and function.

This study demonstrates that AD preconditioning safely induces a reversible state of metabolic suppression in HI, conferring robust protection against severe hypoxia-induced cell death and dysfunction. These findings validate a novel, demand-side cytoprotective strategy with direct applicability to clinical islet transplantation and emerging tissue engineering platforms. AD preconditioning offers a simple, scalable, and highly translatable solution for mitigating early graft loss and enhancing the durability of β-cell replacement therapies for T1D.

## Research in context

### What is already known about this subject?

- Early graft loss after pancreatic islet transplantation is largely driven by severe hypoxia and ischemia–reperfusion injury during the avascular post-transplantation period
- Most current strategies aim to improve islet survival by increasing oxygen supply or accelerating revascularization, with limited clinical success
- Adenosine is an endogenous regulator of cellular metabolism and has been shown in rodent models to suppress insulin secretion, but its effects on human islets under hypoxic stress remain unclear

### What is the key question?

Can adenosine induced reversible metabolic suppression enhance human islet tolerance to hypoxia and preserve viability and function?

### What are the new findings?

- Adenosine safely induces a transient and fully reversible metabolic downregulation in human islets without impairing glucose responsiveness
- Preconditioning human islets with adenosine significantly preserves viability and insulin secretory function following severe hypoxia and reperfusion
- This protection persists after reperfusion and prevents irreversible functional decline associated with hypoxic injury

### How might this impact on clinical practice in the foreseeable future?

Adenosine preconditioning represents a simple, scalable, and translatable “demand-side” strategy that could be readily integrated into clinical islet transplantation and beta-cell replacement workflows to reduce early graft loss.

## Data Availability

The raw data supporting the conclusions of this article will be made available by the authors, without undue reservation.

## Supplementary material

SUPPLEMENTARY FIGURE S1 Long-term Protective effect of AD preconditioning on HI viability and functionality following hypoxia. HIs were preconditioned with 1 mM AD for 24 h and then subjected to 48 h of hypoxia (1% O_2_), followed by 5 days of reperfusion in normoxic medium (21% of O_2_). **(a)** Effect of adenosine before hypoxia exposure on HI viability (N = 3 HI donors; no technical replicates). To conduct GSIS, 100 IEQ were exposed to subsequent glucose concentrations in low glucose (LG1, 2.8 mM), high glucose (HG, 16.7 mM) and LG2 (2.8 mM) solutions. (N = 3, number of different HI donors used and n = 10–11, number of total replicates). **(b)** Insulin content obtained at the end of the GSIS with the exposure to ethanol acid solution. **(c)** Insulin secretion (µg/L). **(d)** Insulin secretion normalized to total insulin content. **(e)** GSIS stimulation index. **(f)** GSIS recovery index. **(g)** GSIS delta of insulin secretion (HG-LG). Data are represented as mean ± SEM, One-way ANOVA with Tukey *post hoc* test, *p < 0.05, **p < 0.01, ***p < 0.001, #p < 0.001, ns, not significant. AD_H, AD treatment for 24 h followed by 48 h of hypoxia; H, hypoxia for 48 h; C, control.

## References

[B1] Antonioli L. Blandizzi C. Csóka B. Pacher P. Haskó G. (2015). Adenosine signalling in diabetes mellitus-pathophysiology and therapeutic considerations. Nat. Rev. Endocrinol. 11, 228–241. 10.1038/nrendo.2015.10 25687993

[B2] Bosco D. Armanet M. Morel P. Niclauss N. Sgroi A. Muller Y. D. (2010). Unique arrangement of alpha- and beta-cells in human islets of langerhans. Diabetes 59, 1202–1210. 10.2337/db09-1177 20185817 PMC2857900

[B3] Cottet-Dumoulin D. Perrier Q. Lavallard V. Matthey-Doret D. Fonseca L. M. Bignard J. (2023). Intercellular contacts affect secretion and biosynthesis of pancreatic islet cells. J. Endocrinol. 258, e220304. 10.1530/JOE-22-0304 37256642

[B4] Dorflinger L. J. Schonbrunn A. (1985). Adenosine inhibits prolactin and growth hormone secretion in a clonal pituitary cell line. Endocrinology 117, 2330–2338. 10.1210/endo-117-6-2330 2998734

[B5] Grunewald M. Avraham I. Dor Y. Bachar-Lustig E. Itin A. Jung S. (2006). VEGF-induced adult neovascularization: recruitment, retention, and role of accessory cells. Cell 124, 175–189. 10.1016/j.cell.2005.10.036 16413490

[B6] Harrison B. S. Eberli D. Lee S. J. Atala A. Yoo J. J. (2007). Oxygen producing biomaterials for tissue regeneration. Biomaterials 28, 4628–4634. 10.1016/j.biomaterials.2007.07.003 17681597

[B7] Hillaire-Buys D. Bertrand G. Gross R. Loubatières-Mariani M. M. (1987). Evidence for an inhibitory A1 subtype adenosine receptor on pancreatic insulin-secreting cells. Eur. J. Pharmacol. 136, 109–112. 10.1016/0014-2999(87)90786-2 3297737

[B8] Holendová B. Benáková Š. Křivonosková M. Plecitá-Hlavatá L. (2024). Redox status as a key driver of healthy pancreatic beta-cells. Physiol. Res. 73, S139–S152. 10.33549/physiolres.935259 38647167 PMC11412338

[B9] Ismail N. A. El Denshary E. E. Montague W. (1977). Adenosine and the regulation of insulin secretion by isolated rat islets of Langerhans. Biochem. J. 164, 409–413. 10.1042/bj1640409 328013 PMC1164806

[B10] Jansson L. Barbu A. Bodin B. Drott C. J. Espes D. Gao X. (2016). Pancreatic islet blood flow and its measurement. Ups. J. Med. Sci. 121, 81–95. 10.3109/03009734.2016.1164769 27124642 PMC4900068

[B11] Johansson S. M. Salehi A. Sandström M. E. Westerblad H. Lundquist I. Carlsson P. O. (2007). A1 receptor deficiency causes increased insulin and glucagon secretion in mice. Biochem. Pharmacol. 74, 1628–1635. 10.1016/j.bcp.2007.08.006 17869224

[B12] Kalka C. Masuda H. Takahashi T. Kalka-Moll W. M. Silver M. Kearney M. (2000). Transplantation of *ex vivo* expanded endothelial progenitor cells for therapeutic neovascularization. Proc. Natl. Acad. Sci. U. S. A. 97, 3422–3427. 10.1073/pnas.070046397 10725398 PMC16255

[B13] Ke D. Murphy S. V. (2019). Current challenges of bioprinted tissues toward clinical translation. Tissue Eng. Part B Rev. 25, 1–13. 10.1089/ten.TEB.2018.0132 30129878

[B14] Ke D. Jorgensen A. M. Lee S. J. Yoo J. J. Murphy S. V. (2021). Adenosine-treated bioprinted muscle constructs prolong cell survival and improve tissue formation. Bio-Des. Manuf. 4, 441–451. 10.1007/s42242-021-00128-5

[B15] Kim J. Andersson K. E. Jackson J. D. Lee S. J. Atala A. Yoo J. J. (2014). Downregulation of metabolic activity increases cell survival under hypoxic conditions: potential applications for tissue engineering. Tissue Eng. Part A 20, 2265–2272. 10.1089/ten.TEA.2013.0637 24524875 PMC4137358

[B16] Koupenova M. Ravid K. (2013). Adenosine, adenosine receptors and their role in glucose homeostasis and lipid metabolism. J. Cell. Physiol., 24352. 10.1002/jcp.24352 23460239 PMC3849123

[B17] Lablanche S. Vantyghem M. C. Kessler L. Wojtusciszyn A. Borot S. Thivolet C. (2018). Islet transplantation versus insulin therapy in patients with type 1 diabetes with severe hypoglycaemia or poorly controlled glycaemia after kidney transplantation (TRIMECO): a multicentre, randomised controlled trial. Lancet Diabetes Endocrinol. 6, 527–537. 10.1016/S2213-8587(18)30078-0 29776895

[B18] Lee H. T. Gallos G. Nasr S. H. Emala C. W. (2004). A1 adenosine receptor activation inhibits inflammation, necrosis, and apoptosis after renal ischemia-reperfusion injury in mice. J. Am. Soc. Nephrol. JASN 15, 102–111. 10.1097/01.asn.0000102474.68613.ae 14694162

[B19] Lee S.-H. Park H. S. Yang Y. Lee E. Y. Kim J. W. Khang G. (2018). Improvement of islet function and survival by integration of perfluorodecalin into microcapsules *in vivo* and *in vitro* . J. Tissue Eng. Regen. Med. 12, e2110–e2122. 10.1002/term.2643 29330944

[B20] Miller J. Perrier Q. Rengaraj A. Bowlby J. Byers L. Peveri E. (2025). State of the art of bioengineering approaches in beta-cell replacement. Curr. Transpl. Rep. 12, 17. 10.1007/s40472-025-00470-y 40342868 PMC12055624

[B21] Ness P. M. Cushing M. M. (2007). Oxygen therapeutics: pursuit of an alternative to the donor red blood cell. Arch. Pathol. Lab. Med. 131, 734–741. 10.1043/1543-2165(2007)131[734:OTPOAA]2.0.CO;2 17488158

[B22] Nichols C. G. Shyng S. L. Nestorowicz A. Glaser B. Clement J. P. Gonzalez G. (1996). Adenosine diphosphate as an intracellular regulator of insulin secretion. Science 272, 1785–1787. 10.1126/science.272.5269.1785 8650576

[B23] Pedraza E. Coronel M. M. Fraker C. A. Ricordi C. Stabler C. L. (2012). Preventing hypoxia-induced cell death in beta cells and islets *via* hydrolytically activated, oxygen-generating biomaterials. Proc. Natl. Acad. Sci. U. S. A. 109, 4245–4250. 10.1073/pnas.1113560109 22371586 PMC3306668

[B24] Perrier Q. Lavallard V. Pernin N. Wassmer C. H. Cottet-Dumoulin D. Lebreton F. (2021). Failure mode and effect analysis in human islet isolation: from the theoretical to the practical risk. Islets 13, 1–9. 10.1080/19382014.2020.1856618 33616002 PMC8018422

[B25] Perrier Q. Cottet-Rousselle C. Lamarche F. Tubbs E. Tellier C. Veyrat J. (2024). Long-term safety of photobiomodulation exposure to beta cell line and rat islets *in vitro* and *in vivo* . Sci. Rep. 14, 26874. 10.1038/s41598-024-77660-8 39505966 PMC11542004

[B26] Perrier Q. Jambon-Barbara C. Kessler L. Villard O. Buron F. Guerci B. (2025). Impact of islet transplantation on diabetes complications and mortality in patients living with type 1 diabetes. Diabetes Care 48, dc250059–dc251015. 10.2337/dc25-0059 40245107 PMC12094206

[B27] Sommerschild H. T. Kirkebøen K. A. (2000). Adenosine and cardioprotection during ischaemia and reperfusion-an overview. Acta Anaesthesiol. Scand. 44, 1038–1055. 10.1034/j.1399-6576.2000.440903.x 11028722

[B28] Szkudelski T. Szkudelska K. (2015). Regulatory role of adenosine in insulin secretion from pancreatic β-cells-action via adenosine A_1_ receptor and beyond. J. Physiol. Biochem. 71, 133–140. 10.1007/s13105-014-0371-y 25432862

[B29] Toso C. Zaidi H. Morel P. Armanet M. Andres A. Pernin N. (2005). Positron-emission tomography imaging of early events after transplantation of islets of Langerhans. Transplantation 79, 353–355. 10.1097/01.tp.0000149501.50870.9d 15699768

[B30] Wojtusciszyn A. Branchereau J. Esposito L. Badet L. Buron F. Chetboun M. (2019). Indications for islet or pancreatic transplantation: statement of the TREPID working group on behalf of the Société francophone du diabète (SFD), Société francaise d’endocrinologie (SFE), Société francophone de transplantation (SFT) and Société française de néphrologie - dialyse - transplantation (SFNDT). Diabetes Metab. 45, 224–237. 10.1016/j.diabet.2018.07.006 30223084

[B31] Yang J. Shi G. Bei J. Wang S. Cao Y. Shang Q. (2002). Fabrication and surface modification of macroporous poly(L-lactic acid) and poly(L-lactic-co-glycolic acid) (70/30) cell scaffolds for human skin fibroblast cell culture. J. Biomed. Mater. Res. 62, 438–446. 10.1002/jbm.10318 12209930

[B32] Zywert A. Szkudelska K. Szkudelski T. (2011). Effects of adenosine A(1) receptor antagonism on insulin secretion from rat pancreatic islets. Physiol. Res. 60, 905–911. 10.33549/physiolres.932165 21995904

