# Supplementary material for: Adenosine as a modulator of human islet function and hypoxic tolerance
Source: Front Bioeng Biotechnol. 2026 Jun 1;14:1828405. doi: 10.3389/fbioe.2026.1828405 (PMC13266289; doi:10.3389/fbioe.2026.1828405)
Supplement: Supplementary file 1 [file Supplementaryfile1.docx]

**Figure S1. Long-term Protective effect of AD preconditioning on HI viability and functionnality following hypoxia**


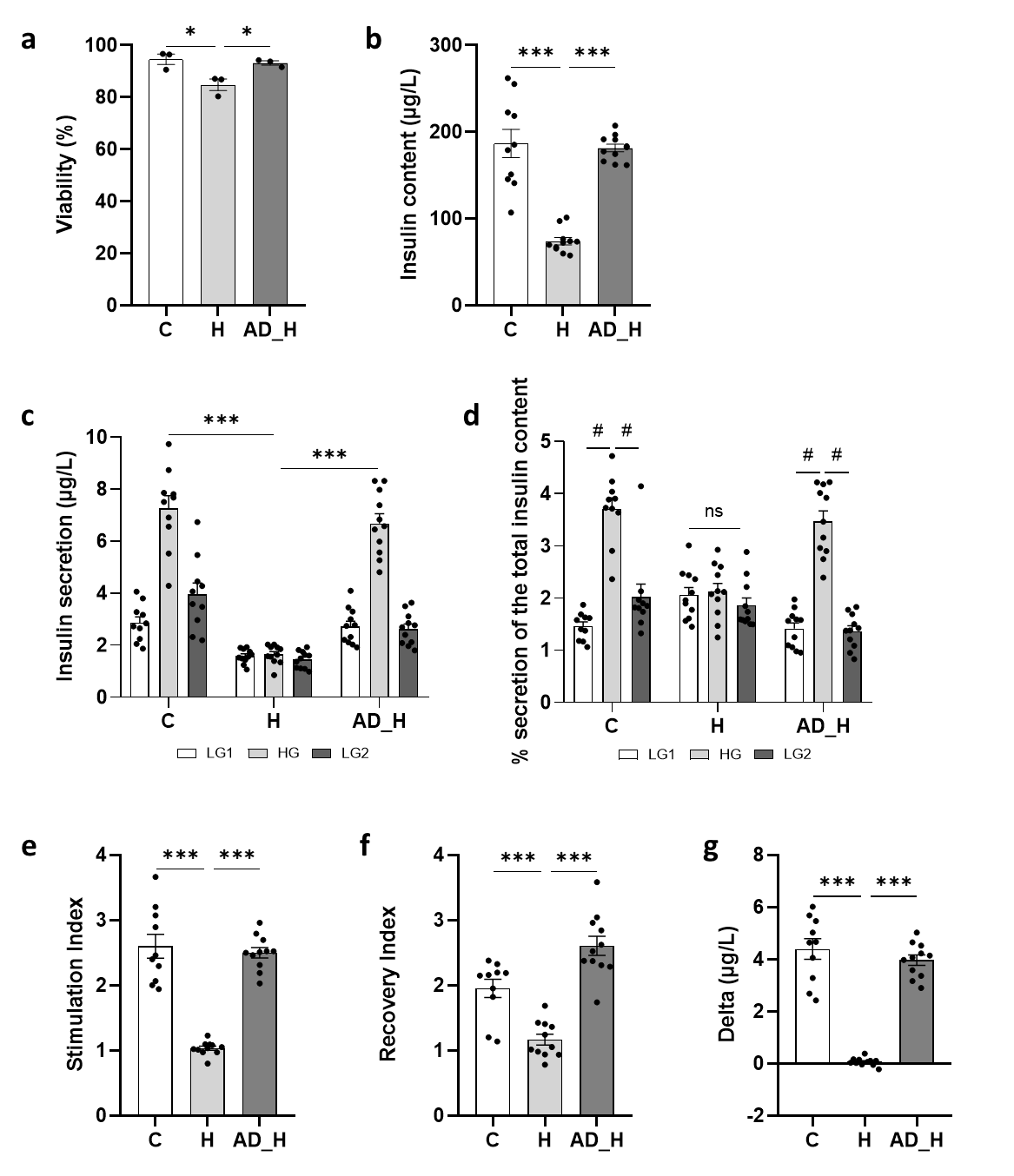


HIs were preconditioned with 1 mM AD for 24h and then subjected to 48h of hypoxia (1% O_2_), followed by 5 days of reperfusion in normoxic medium (21% of O_2_). **(a)** Effect of adenosine before hypoxia exposure on HI viability (N = 3 HI donors; no technical replicates). To conduct GSIS, 100 IEQ were exposed to subsequent glucose concentrations in low glucose (LG1, 2.8 mM), high glucose (HG, 16.7 mM) and LG2 (2.8 mM) solutions. (N = 3, number of different HI donors used and n = 10-11, number of total replicates). **(b)** Insulin content obtained at the end of the GSIS with the exposure to ethanol acid solution. **(c)** Insulin secretion (µg/L). **(d)** Insulin secretion normalized to total insulin content. **(e)** GSIS stimulation index. **(f)** GSIS recovery index. **(g)** GSIS delta of insulin secretion (HG-LG). Data are represented as mean ± SEM, One-way ANOVA with Tukey post-hoc test, * p<0.05, ** p<0.01, *** p<0.001, # p<0.001, ns – not significant. AD_H: AD treatment for 24h followed by 48h of hypoxia, H: hypoxia for 48h, C: control.
